# Micro-structured polyethylene film as an optically selective and self-cleaning layer for enhancing durability of radiative coolers

**DOI:** 10.1515/nanoph-2023-0198

**Published:** 2023-05-05

**Authors:** Yi Jiang, Jiahao Wang, Yaya Zhou, Jinlei Li, Zipeng Chen, Pengcheng Yao, Haixiong Ge, Bin Zhu

**Affiliations:** National Laboratory of Solid State Microstructures, College of Engineering and Applied Sciences, Jiangsu Key Laboratory of Artificial Functional Materials, Collaborative Innovation Center of Advanced Microstructures, Nanjing University, Nanjing 210023, P.R. China

**Keywords:** infrared transparent, micro-structured polyethylene film, radiative cooling, self-cleaning

## Abstract

Passive daytime radiative cooling (PDRC) as a zero-energy cooling technology that reflects most of sunlight and emits infrared thermal radiation to outer space, has attracted much attention. However, most PDRC materials suffer dust accumulation problem during long-term use, seriously detrimental to their cooling performance. Here, we demonstrate a micro-structured polyethylene film fabricated through a scalable hot embossing lithography (named HELPE), enables good superhydrophobic property and therefore excellent self-cleaning performance as a universal protective layer for most PDRC materials. Specifically, the precisely designed three-dimensional periodic micron columns on polyethylene film allow for high water droplet contact angle of 151°, and the intrinsic molecular bindings of polyethylene endow low solar absorption (*A* = 3.3 %) and high mid-infrared transmission (*T* = 82.3 %) for negligible optical impacts on underlying PDRC materials. Taking polyvinylidene fluoride (PVDF) radiative cooler as an example, when covered with the HELPE film the net cooling performance maintains unchanged (7.5 °C in daytime and 4.5 °C in nighttime) compared to that without HELPE film. After 12 days continuous outdoor experiment, none of obvious dust accumulation can be observed on the radiative cooler covered with HELPE film. Our work offers a universal pathway for most PDRC materials toward practical applications with minimal maintenance need.

## 1 Introduction

**Figure 1:**
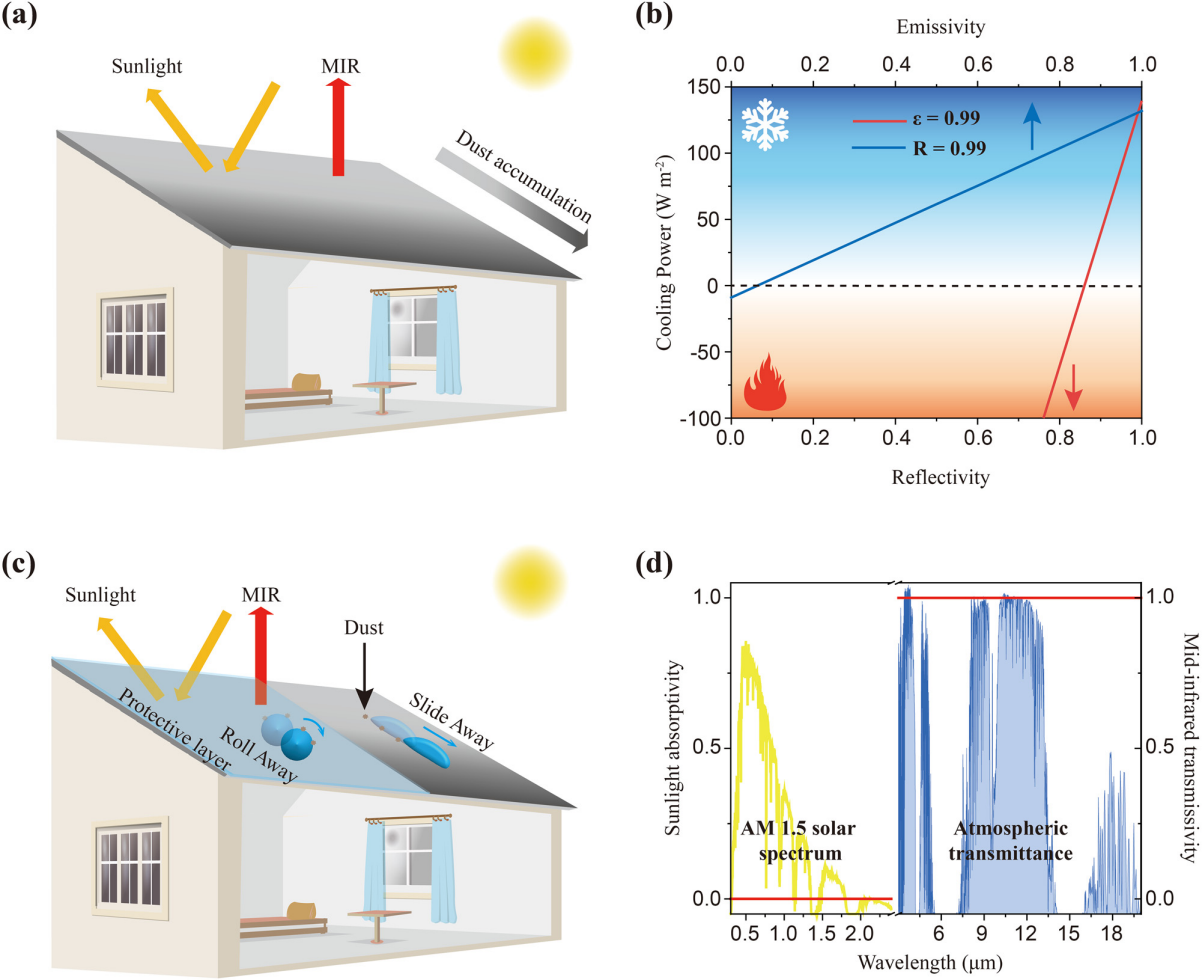
Design of the protective cover. (a) Dust accumulation problem of most PDRC materials. The yellow and red arrows represent solar reflection and heat radiation, respectively. (b) Cooling power versus reflectivity (or emissivity) when assuming emissivity (or reflectivity) as 0.99 at an ambient temperature of 25 °C. (c) The design of a protective layer, with superhydrophobic property and weak optical influence on PDRC materials, is able to alleviate dust problem. (d) Ideal spectrum of the protective layer.

As conventional refrigeration methods such as air conditioner and fan lead to tremendous electricity consumption and carbon emission, it is desirable to develop energy-saving cooling strategies [1, 2]. Passive daytime radiative cooling (PDRC), which reflects most of sunlight (0.3–2.5 μm) and emits infrared thermal radiation into the outer space (∼3 K) through atmospheric transparency window (8–13 μm), can effectively cool objects without energy consumption [3], [4], [5], [6], [7], [8]. Recently, various PDRC materials such as poly(vinylidene fluoride-co-hexafluoropropene), polyethylene oxide, nanoprocessed silk, and cellulose acetate, have been proposed and shown excellent cooling performance [9], [10], [11], [12], [13], [14], [15], [16], [17]. However, most PDRC materials suffer durability problems in the practical applications [18, 19], including the dust accumulation (Figure 1a). As is well-known, to enhance the cooling performance, the PDRC materials always appear white or silver to maximize the reflectance of sunlight [20], [21], [22]. When dust aggregates on the surface of PDRC materials working outdoor, the solar reflection inevitably decrease due to the sunlight absorption of dust [23], significantly affecting their cooling performance (Figure 1b, detailed in Supporting Information) [24]. Some research has focused on regulating structures together with components of PDRC materials to address this problem, but they always increase the complexity of design and production [25], [26], [27]. Essentially, creating a protective layer to isolate or get rid of dust from PDRC materials can be a universal strategy for improving their durability in practical applications.

The ideal protective layer not only needs to possess good self-cleaning property to remove dust accumulation, but also does not affect the intrinsic optical properties of underlying PDRC materials (Figure 1c). Specifically, it first should be superhydrophobic (water contact angle >150°) because the previous research indicated that the water droplet rolls away on the superhydrophobic layer rather than slides away or even spreads out, which will remove dust more effectively [28], [29], [30], [31], [32], [33]. In addition, the protective layer must present a low solar absorption and a high mid-infrared transmission to minimize the optical influence for the PDRC materials (the ideal spectrum illustrated in Figure 1d). Polyethylene (PE), which has only C–C and C–H bonds and shows low solar absorption and high mid-infrared transmission as reported before, may be a candidate for the protective layer [34], [35], [36], [37], [38]. However, there exists difficulty to make PE superhydrophobic without any auxiliary materials that may introduce new molecular vibration mode.

Herein, we demonstrate a micro-structured polyethylene film through hot embossing lithography (HELPE), enables both superhydrophobic property and low optical absorption across 0.3–20 μm as a universal protective layer for PDRC. The three-dimensional periodic micron columns on the HELPE film allow for water contact angle of 151° without sacrificing cooling performance of PDRC materials due to the low solar absorption of 3.3 % and the high mid-infrared transmission of 82.3 %. As a demonstration with polyvinylidene fluoride (PVDF) film as the radiative cooler, we observe similar cooling temperature (7.5 °C in daytime and 4.5 °C in nighttime) and great antifouling effect when covered with the HELPE film, compared with PVDF film alone. It is expected that this work offers a universal protection strategy for most PDRC materials and paves a promising way to speed the practical applications of PDRC with minimal maintenance requirement.

## 2 Fabrication and characterizations of the HELPE film

**Figure 2:**
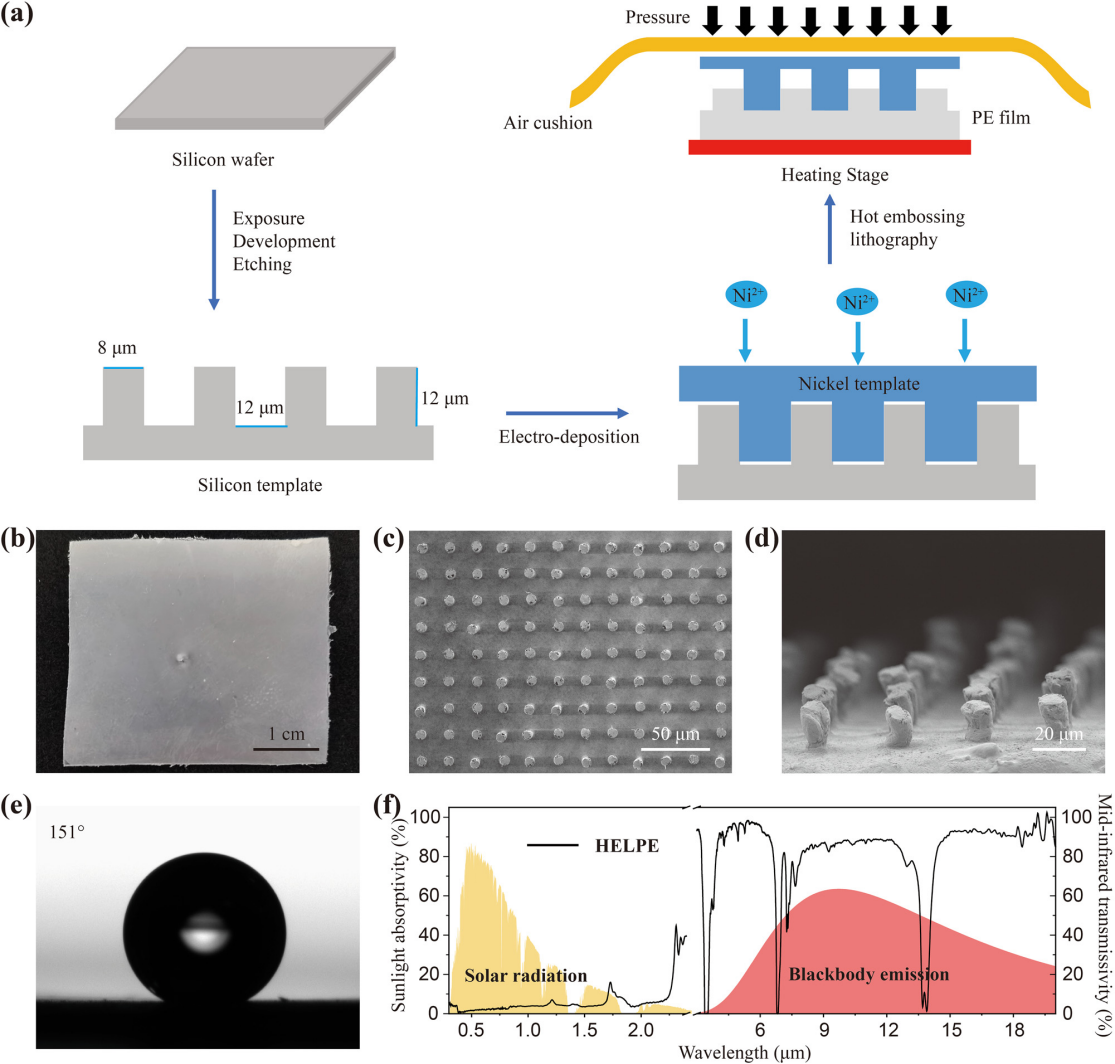
Fabrication and characterizations of the HELPE film. (a) Fabrication process of the HELPE film. (b) Photograph of the fabricated HELPE film. (c, d) SEM images of the top surface and the cross-section of the HELPE film, respectively. (e) Water droplet contact angle of the HELPE film. (f) Absorption spectrum of the HELPE film against solar radiation and transmission spectrum against blackbody emission in 0.3–20 μm wavelength.

Modern hot embossing lithography enables great probability for the scalable fabrication of the HELPE film by realizing three-dimensional periodic structure on the PE film (Figure 2a, detailed in Supporting Information). The optical photograph of the final HELPE film is shown in Figure 2b.

It is important to first verify the successful construction of microstructure on the HELPE film. The scanning electron microscope (SEM) bird’s view in Figure 2c shows periodic micron columns with a diameter of 7.73 μm and a period of 19.76 μm and the cross-sectional view in Figure 2d shows columns with height of 11.45 μm. The obtained microstructures match well with the designed value, and is expected to have an excellent hydrophobic effect. As shown in Figure 2e, the water droplet contact angle on the surface of the HELPE film is measured to be 151°, which is significantly larger than that of the intrinsic PE of 100° (Figure S1, Supporting Information) and beneficial to the self-cleaning performance.

We subsequently monitor the optical properties of the HELPE film in the solar spectrum and mid-infrared band (Figure 2f). It is clearly seen that the absorption of the HELPE in the solar spectrum is only 3.31 % and transmission in mid-infrared band reaches 82.38 %, thereby suggesting the potential of this film as a universal protective layer for most PDRC materials with minimal impact on optical properties.

With better selection of variable molds instead of the silicon wafer, the size of the HELPE film fabricated from single-step pattern transfer through hot embossing lithography can be much larger. Combined with the developing roll-to-roll and roll-to-plane nanoimprint lithography, it is promising to produce large-area sample of such photonic structures [39, 40].

## 3 Performance test of the HELPE film

**Figure 3:**
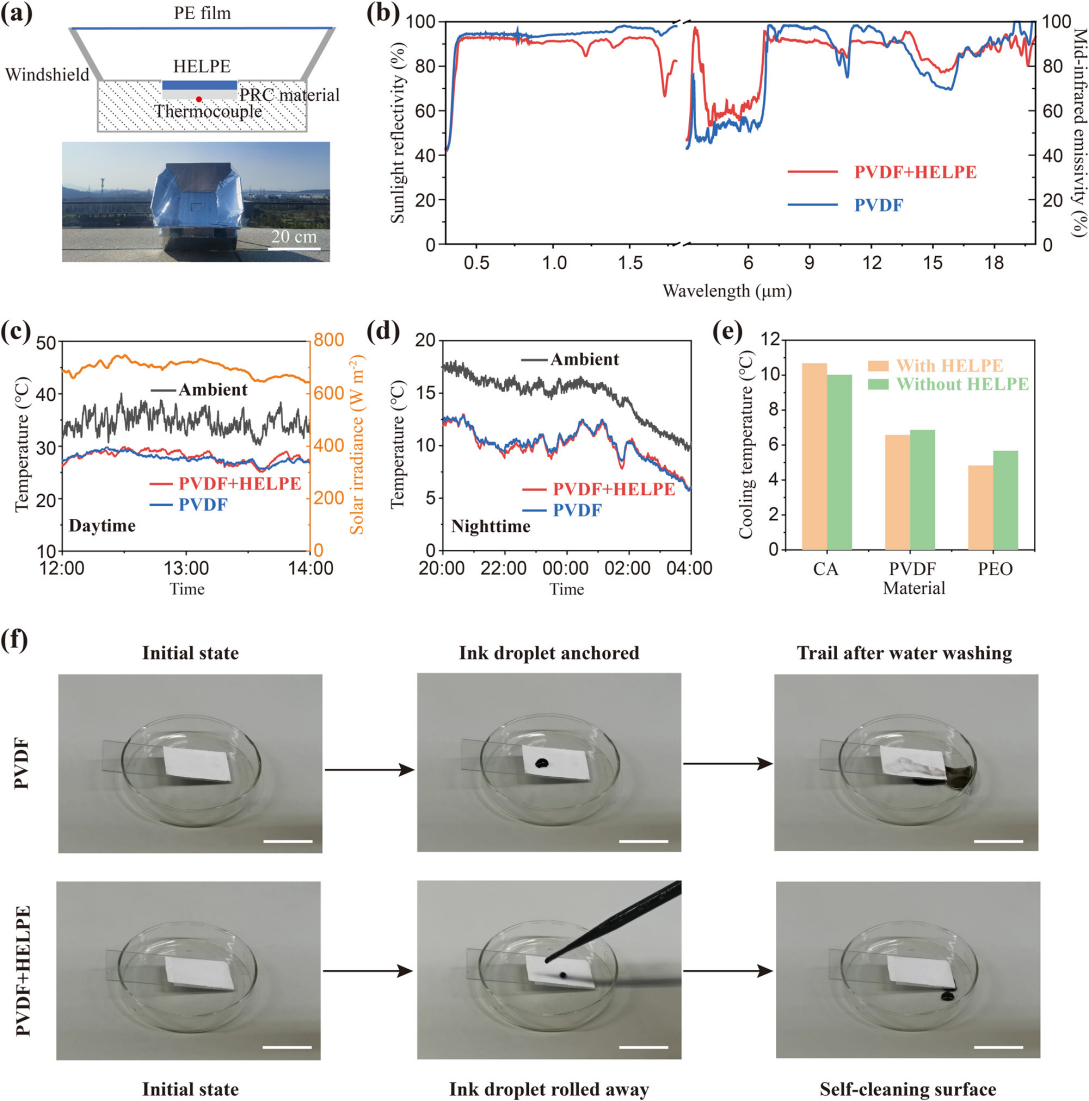
Performance test of the HELPE film. (a) Schematic and photograph of the setup for testing radiative cooling performance. (b) The optical spectra of the PVDF film covered with and without the HELPE film. (c) Temperature comparisons between PVDF films covered with and without the HELPE film under 700 W m^−2^ of solar radiation. (d) Nighttime temperature comparisons in the PVDF film covered with and without the HELPE film. (e) Cooling temperature of different PDRC materials (CA, PVDF, and PEO) covered with and without the HELPE film, respectively. (f) The antifouling performance of the HELPE film rinsed by black ink (Scale bar: 3 cm).

It is necessary to experimentally compare the cooling performance of PDRC materials with and without the HELPE film to further prove the negligible optical impacts of the HELPE film on PDRC materials. We perform outdoor temperature measurement with both PVDF film covered with HELPE film and PVDF film alone in Nanjing, using the setups shown in Figure 3a. The setup is insulated from heat conduction by foam and convection by PE film, respectively, to minimize the thermal impacts from ambient. The optical spectra of the two samples across 0.3–20 μm are plotted in Figure 3b with similar solar reflection and mid-infrared emission. The average reflectivity over the solar spectrum of the PVDF film covered with the HELPE film is 90.4 % and that of the PVDF film alone is 94.1 %. Simultaneously, the average mid-infrared emissivity of the PVDF film covered with the HELPE film is 86.7 % and that of the PVDF film alone is 86.6 %. Under a sunlight intensity of ∼700 W m^−2^ (from 12:00 to 14:00), it is observed that the PVDF films with and without the HELPE film has similar temperatures (with a difference within 0.5 °C) and both enables a subambient cooling temperature of about 7.5 °C (Figure 3c). Additionally, the temperature measurement is also conducted in the nighttime in order that the impacts of sunlight are excluded and only the optical property of mid-infrared band is studied. It is obvious that the temperatures of both samples again stay the same and are 4.5 °C cooler than ambient temperature from 20:00 to next 4:00 (Figure 3d and Figure S2, Supporting Information). All the results consistently suggest that the introduction of the HELPE film has negligible impacts on the cooling performance of the PDRC materials.

In addition to the PVDF film, we perform cooling performance test on other PDRC materials, such as cellulose acetate (CA) and polyethylene oxide (PEO) films. As shown in Figure 3d, whether in daytime or nighttime (Figures S3, 4, and 5, Supporting Information), the cooling temperatures of different PDRC materials with and without the HELPE film have negligible difference. Therefore, the HELPE film can serve as a universal protective layer for most PDRC materials.

Next, we examine the antifouling performance of the HELPE film under a simulated environment. Black ink is employed to contaminate the surface of the PVDF films with and without the HELPE film (Figure 3f). When the ink droplet falls onto the surface of the PVDF film, it is anchored to the surface, requiring purified water to wash it away. Simultaneously, the trail of rinsed droplets makes the surface dark, resulting in reduced reflectivity in the solar spectrum. Whereas, on the surface of the PVDF film with the HELPE film, the falling ink droplet immediately rolls off and has no contamination stains. This is attributed to the excellent superhydrophobic properties of surface micro-structure on the HELPE film.

The cooling performance and the antifouling tests together confirm that the HELPE film is an effective protective layer for PDRC materials.

## 4 Outdoor tests

**Figure 4:**
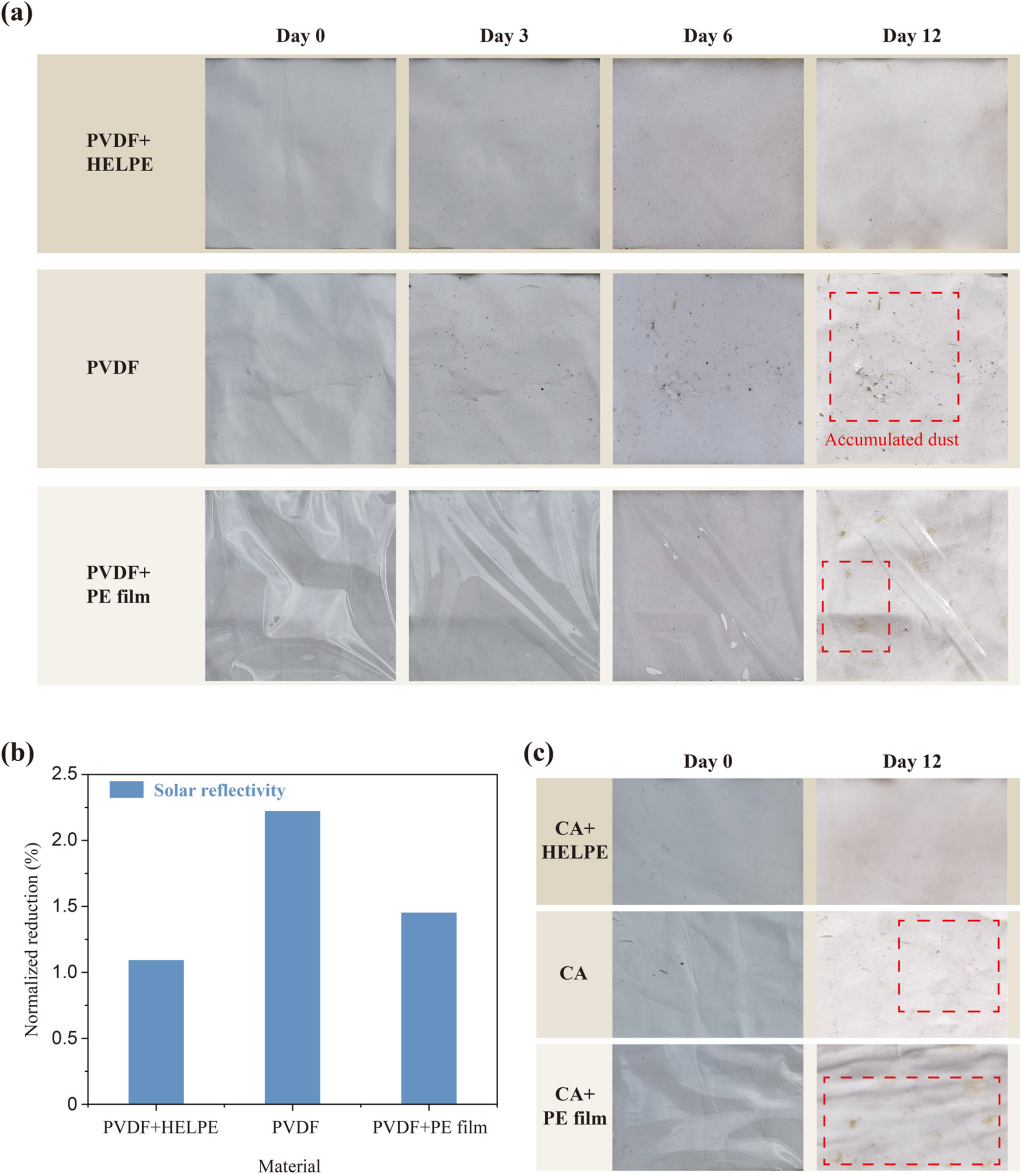
Outdoor tests. (a) Photographs record the surface changes of the PVDF film covered with the HELPE film, the PVDF film alone and the PVDF film with PE film without micro-structures during the 12 days of continuous outdoor exposure. The samples are all sized 25 cm^2^. The dotted red frame highlights the accumulated dust. (b) Normalized reduction in solar reflectivity of the samples. (c) Antifouling performance universality test of the HELPE film on the CA film outdoors.

To demonstrate the antifouling performance of the HELPE film under outdoor conditions, the PVDF film covered with the HELPE film, the PVDF film alone, and the PVDF film covered with PE film without micro-structures are exposed outdoors for a continuous 12-day test in Nanjing. The photographs in Figure 4a record the dust changes on the surface. It is founded that the PVDF film covered with the HELPE film stays clean. In comparison, the PVDF film covered with PE film and PVDF film alone became increasingly dirty with dust accumulation. The contrast is attributed to the better self-cleaning property of the HELPE film, compared with PE film and PVDF film. Although the three samples are subjected to similar environmental dust in daytime, in nighttime, congealed dew caused by subambient cooling of PDRC materials help the PDRC film under the protection of the HELPE film with excellent hydrophobicity remove more surface dust. We measure the optical properties of three samples in both the solar and mid-infrared wavebands on the sixth day of outdoor exposure and normalize the data based on initial spectrum test. As shown in Figure 4b, the PVDF film covered with the HELPE film has minimal reduction of solar reflectivity (1.09 %), which is 0.36 % smaller than that of the PVDF film covered with PE film and is just half of the PVDF film (2.22 %), suggesting that there is the least dust on its surface and the HELPE film can help PDRC materials maintain the cooling performance. On the other hand, the mid-infrared emissivity also has minimal increase on the PVDF film covered with the HELPE film (1.11 %), which indirectly verifies the slightest impact by dust (Figures S6 and 7, Supporting Information). Other than the PVDF film, the antifouling performance of the HELPE film is also verified on CA radiative cooling films during a continuous 12-day test (Figure 4c), further illustrating the universality of the HELPE film.

## 5 Conclusions

In summary, we report a micro-structured HELPE film with superhydrophobic and optically selective properties to act as an effective and universal protective layer for most PDRC materials without sacrificing its intrinsic cooling performance. As a demonstration with PVDF radiative cooler, its cooling temperature remains unchanged when covered with the HELPE film, both in daytime and nighttime. Importantly, the HELPE film prevents pollutants including ink and dust from staining the clean surface of the PVDF film and thus leading to the detrimental degradation of sunlight reflectivity due to its super-hydrophobicity. Our work provides a new pathway to speed the durable applications for most PDRC materials.
